# Staff Changes at Claybury

**Published:** 1914-02-21

**Authors:** Robert Armstrong-Jones

**Affiliations:** Resident Physician and Superintendent. Claybury Asylum, Woodford Bridge, Essex


### Staff Changes at Claybury.

*To the Editor of* The Hospital.

Sir,—The Hospital, usually fair in its criticism and accurate in its information, has, in the issue of February 7, a paragraph on p. 496 headed "Staff Changes at Claybury Hospital," stating therein "that, despite the improvements which have recently been made in the conditions of service, the Mental Hospitals Committee of the London County Council is still finding a difficulty in filling up the ranks of its assistant medical officers."

May I state that this is not quite correct? When Mr. O. H. Bowen left in the summer of 1913 to become a dental student, the medical officers entering subsequent to him all received promotion, and Dr. D. W. Rintoul became the junior medical officer temporarily. He has since passed seventh into the R.A.M.C., for which he was destined. Later one of his colleagues, Dr. E. G. T. Poynder, was promoted to be a deputy to the medical superintendent of the Egyptian Lunatic Asylum in Cairo, after good and efficient services duly recognised by the Claybury Committee. I may state here that this makes the second appointment of a deputy, where Dr. Warnock in Cairo has two of the medical staff of Claybury as his colleagues, and this in addition to three nurses also from Claybury, who recently left to be assistant matrons in that asylum.

The two junior medical posts thus occurring in Claybury were filled up without any delay, two well-qualified gentlemen joining the service when required, and several applications having also been received since, one on behalf of "the best cricketer in Ireland," who, I may add, had, in addition, other more serious qualifications.

Your comments imply a meticulous emotion in regard to joining the London Asylum Medical Service, whereas the reverse is the case; at any rate, so far as the Claybury Hospital is concerned. Promotion among the staff is always taken to be a healthy symptom and a sign that services well rendered are duly appreciated. It is also an inducement for others to join in the hope of a like preferment. —I am, Sir, your obedient servant,

Robert Armstrong-Jones, M.D.,
Resident Physician and Superintendent.

Claybury Asylum,
Woodford Bridge, Essex.
February 11, 1914.

